# Supplementary material for: Insight into Tetramolecular DNA G-Quadruplexes Associated with ALS and FTLD: Cation Interactions and Formation of Higher-Ordered Structure
Source: Int J Mol Sci. 2023 Aug 30;24(17):13437. doi: 10.3390/ijms241713437 (PMC10487854; doi:10.3390/ijms241713437)
Supplement: Supplementary file 1 [file ijms-24-13437-s001.zip › ijms-2538586-supplementary.pdf]

Supplementary information for:

Insight into tetramolecular DNA G-quadruplexes associated with ALS and FTLD: Cation interactions and formation of higher ordered structure

Matja Zalar[1], Baifan Wang[1], Janez Plavec[1,2,3] and Primož Šket[1,*]

[1] Slovenian NMR Center, National Institute of Chemistry, Hajdrihova 19, SI-1000 Ljubljana, Slovenia
[2] EN-FIST Center of Excellence, Trg OF 13, SI-1000 Ljubljana, Slovenia
[3] Faculty of Chemistry and Chemical Technology, University of Ljubljana, Večna pot 113, SI-1000 Ljubljana, Slovenia
* Correspondence: primoz.sket@ki Tel: +386 1 4760223; Fax: +386 1 4760300

SUPPLEMENTARY FIGURES

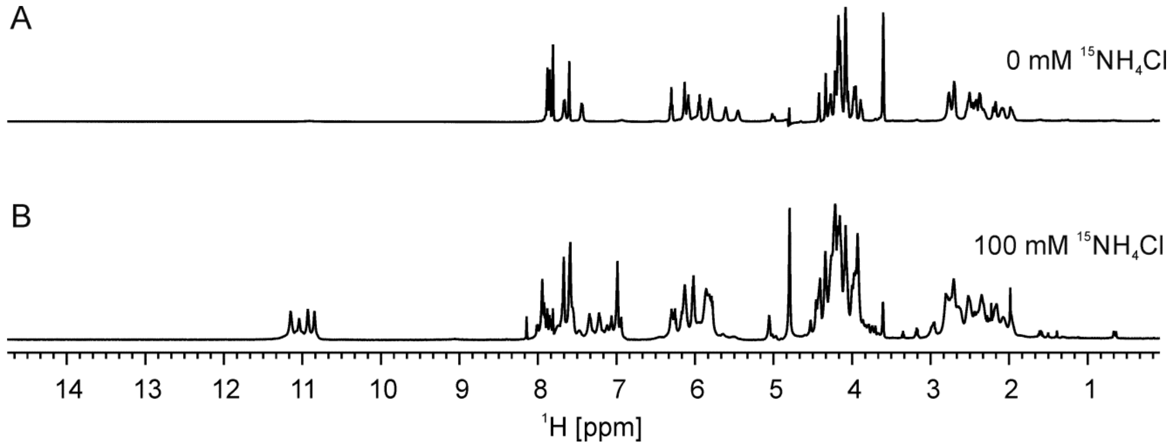

Figure S1: ^{1}H NMR spectrum of $d(G_4C_2)$ A) before and B) after addition of 100 mM $^{15}NH_4^+$ ions. Both spectra were recorded in 10% of ^2H_2O on 600 MHz NMR spectrometer at 25 °C, pH 6.0. Oligonucleotide concentration was 2.0 mM.

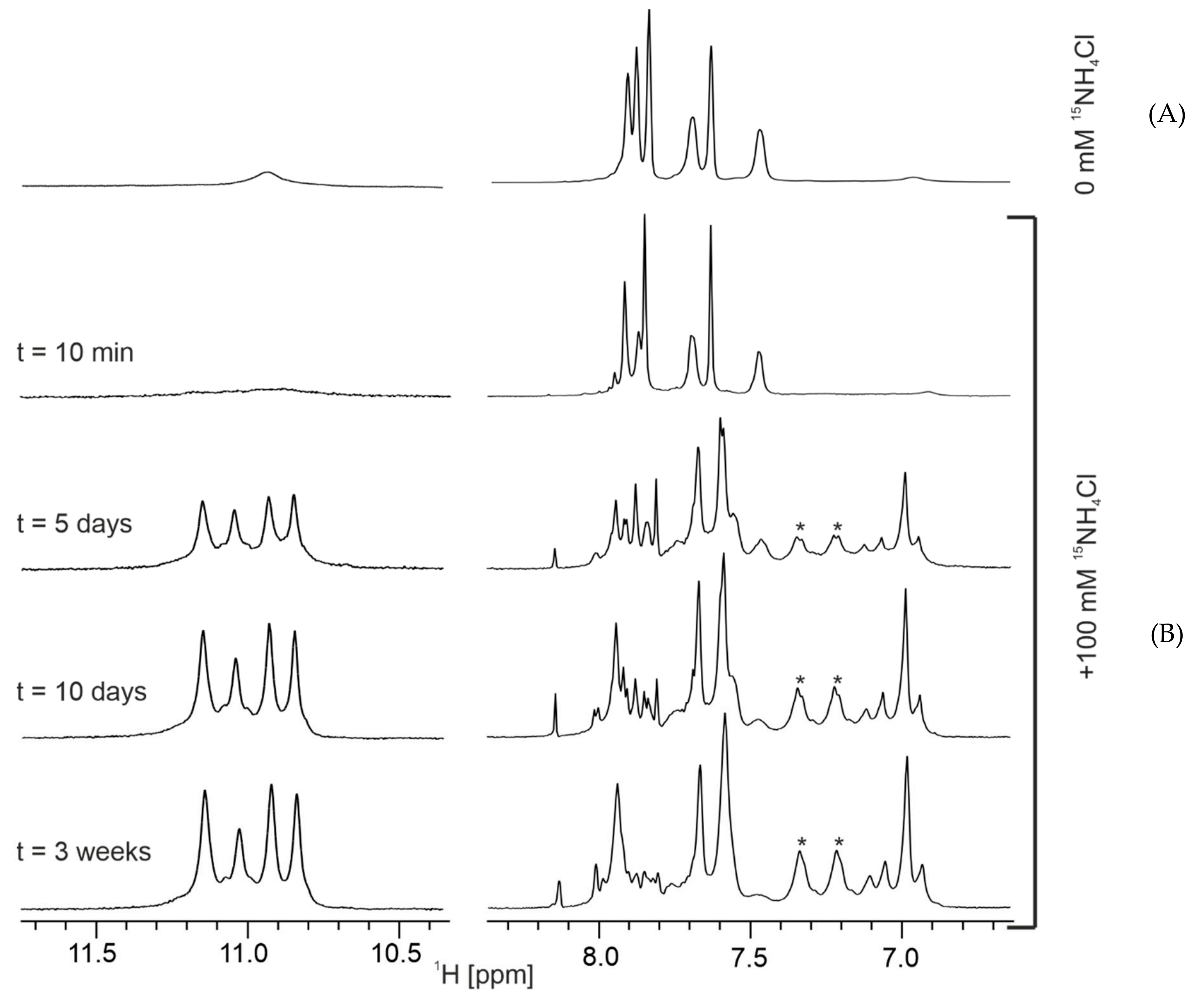

Figure S2: Imino (A) and aromatic (B) regions of ^{1}H NMR spectra of oligonucleotide d(G_4C_2) before and after addition of 100 mM $^{15}NH_4^+$ ions concentration as a function of time. Intensities in A were enlarged two times in respect to other signals. Asterisks denote signals of ammonium ions at binding site O_5. All spectra were recorded in 10% of ^2H_2O at 25°C, pH 6.0 on 600 MHz NMR spectrometer. Oligonucleotide concentration was 2.0 mM.

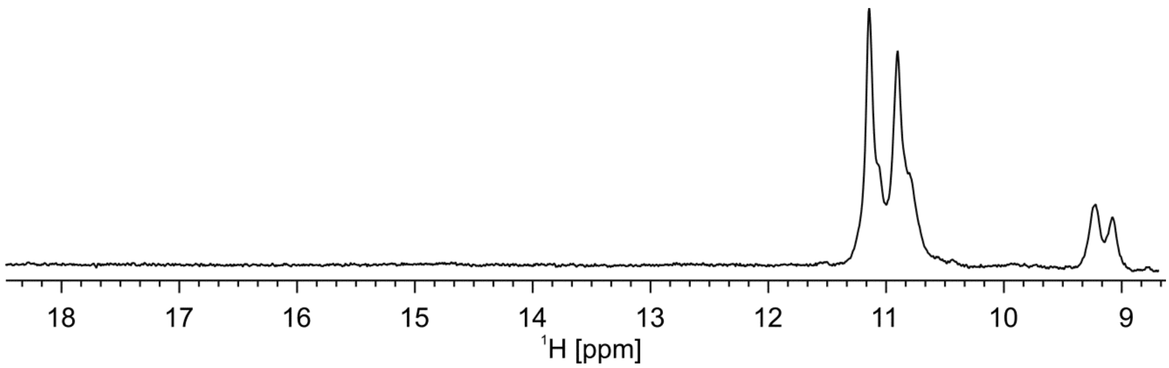

Figure S3: Imino region of ^{1}H NMR spectrum of $[d(G_4C_2)]_4$ G-quadruplex in the presence of 100 mM $^{15}NH_4Cl$ at 5 °C, pH 4.5. Spectrum was recorded on 600 MHz NMR spectrometer at 2 mM oligonucleotide concentration.

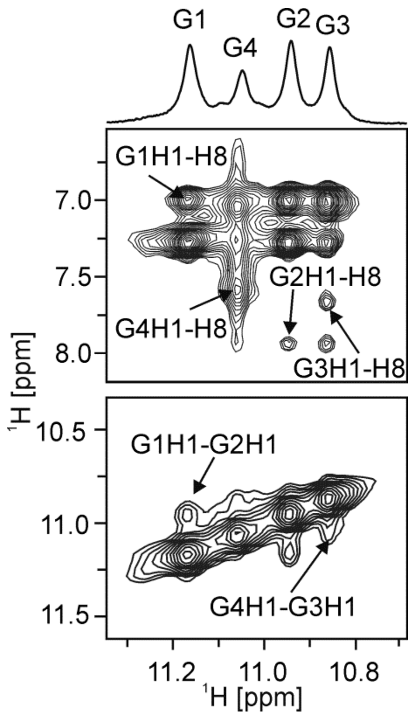

Figure S4: Imino -aromatic and imino-imino regions of ^{15}N decoupled NOESY spectrum (τ_m= 150 ms) of $[d(G_4C_2)]_4$. Assignments are shown next to corresponding cross-peaks. Imino region of ^{1}H spectrum is shown on top together with signal assignments. Spectrum was recorded on 600 MHz NMR spectrometer, 25 °C in 90% H_2O and 10% $^{2}H_2O$, 100 mM $^{15}NH_4Cl$, pH 6.0. Oligonucleotide concentration was 2 mM.

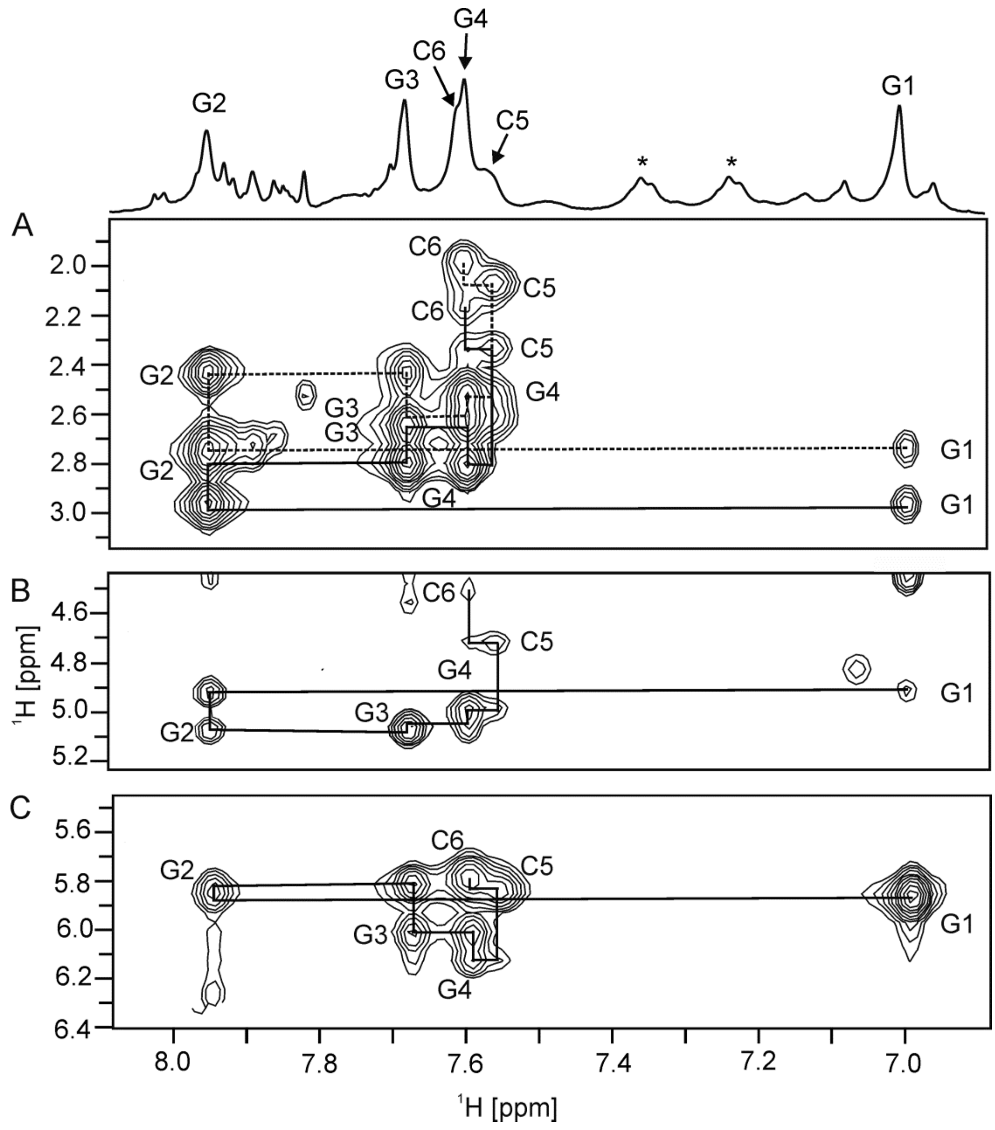

Figure S5: The aromatic H6/H8 –sugar H2′/H2′′ (A), H3′ (B) and H1′ (C) regions of NOESY spectrum (τ_m= 150 ms) of [d(G_4C_2)]$_4$. All sequential walks except H8/H6-H2′, which is shown with dashed black line, are depicted in solid black lines. Assignments are shown next to corresponding cross-peaks. Aromatic region of ^{1}H NMR spectrum is shown on top together with signal assignments. Signals labeled with stars correspond to $^{15}NH_4^+$ ions at binding site O_5. Spectrum was recorded on 600 MHz NMR spectrometer, 25 °C in 90% H_2O and 10% ^2H_2O, 100 mM $^{15}NH_4Cl$, pH 6.0. Oligonucleotide concentration was 2 mM.

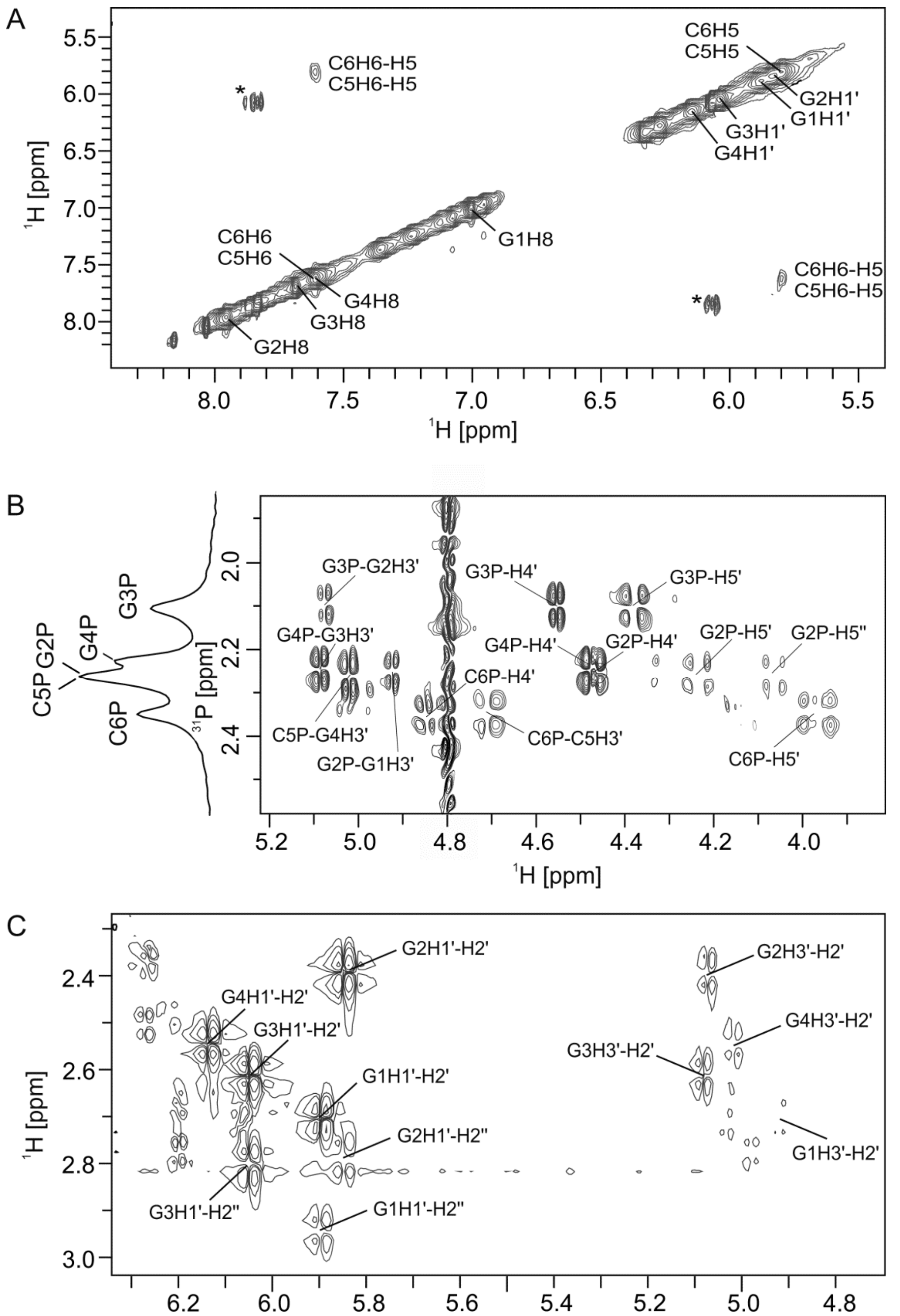

Figure S6: A) Aromatic and anomeric regions of TOCSY spectrum (τ_m=20 ms) of [d(G_4C_2)]$_4$. B) ^{1}H-^{31}P COSY spectrum of [d(G_4C_2)]$_4$ with ^{31}P NMR spectrum shown on the left hand side. C) Sugar H1′/H3′ – sugar H2′/H2′′ region of DQF-COSY spectrum of [d(G_4C_2)]$_4$. Spectra were recorded on 600 MHz NMR spectrometer, 25 °C in 100 % D_2O, 100 mM $^{15}NH_4Cl$, pH 6.0. Oligonucleotide concentration was 2.0 mM. Asterisks in A denote organic impurity in the sample. Assignments are indicated next to the cross-peaks.

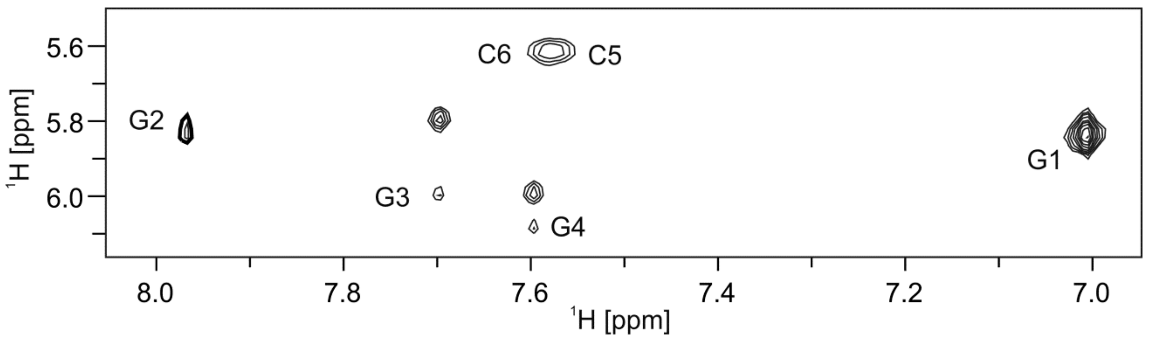

Figure S7: The aromatic H6/H8 – anomeric H1′ region of NOESY spectrum (τ_m= 80 ms) of [d(G_4C_2)]$_4$. Assignments are shown next to corresponding cross-peaks. Stronger intensity of G1H8-H1′ in comparison to cross-peaks corresponding to other residues indicates *syn* orientation around glycosidic bond for G1. Spectrum was recorded on 600 MHz NMR spectrometer, 25 °C in 100% ^2H_2O, 100 mM $^{15}NH_4Cl$, pH 6.0. Oligonucleotide concentration was 2 mM. Note that chemical shifts of C5H6 and C6H6 depend on ^2H_2O concentration.

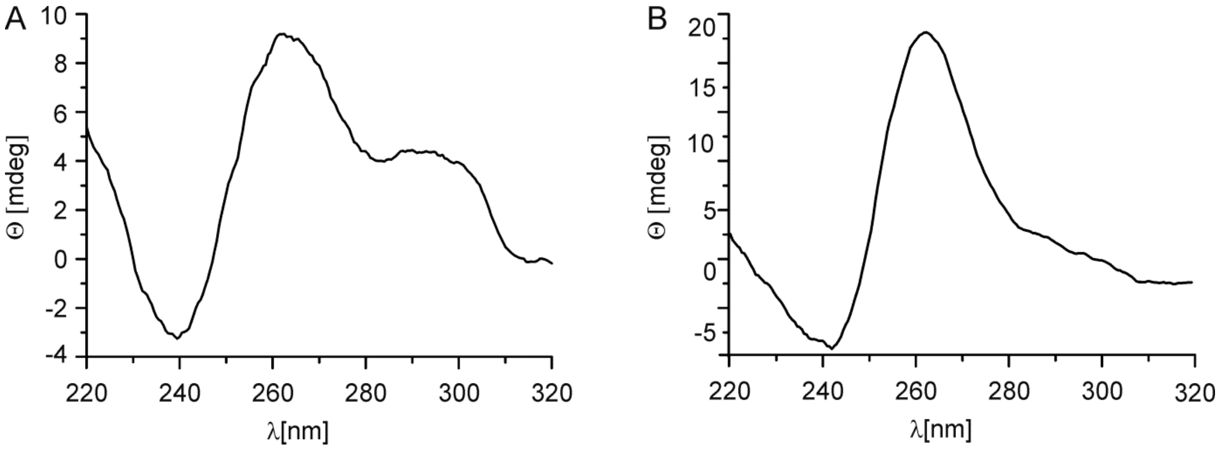

Figure S8: A) CD spectrum of oligonucleotide d(G_4C_2) in presence of 100 mM $^{15}NH_4Cl$. B) CD spectrum of oligonucleotide d(G_4C_2) in presence of 100 mM $^{15}NH_4Cl$ and 80 mM KCl. Both spectra were recorded at 2mM concentration of d(G_4C_2) in 10 mM Li-Cacodylate buffer, pH 6.0 at 25 °C.

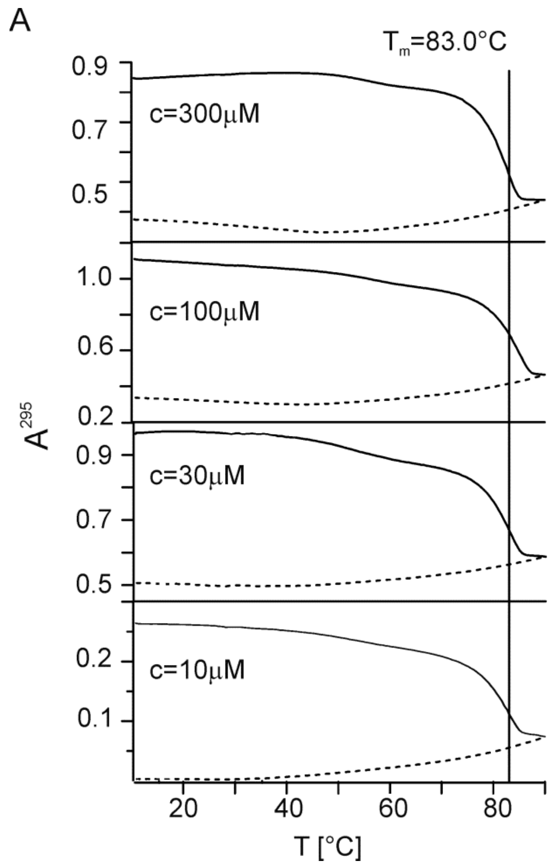

Figure S9: A) Normalized UV melting and annealing curves of d(G_4C_2) oligonucleotide in the presence of 100 mM $^{15}NH_4Cl$ obtained at 295 nm. B) Normalized UV melting and annealing curves of d(G_4C_2) oligonucleotide in the presence of 80 mM KCl and 100 mM $^{15}NH_4Cl$ at 295 nm. Spectra were recorded in 10 mM Li-cacodylate buffer, pH 6.0. Oligonucleotide concentrations are indicated next to the spectra. Concentration of oligonucleotide is marked on each plot.

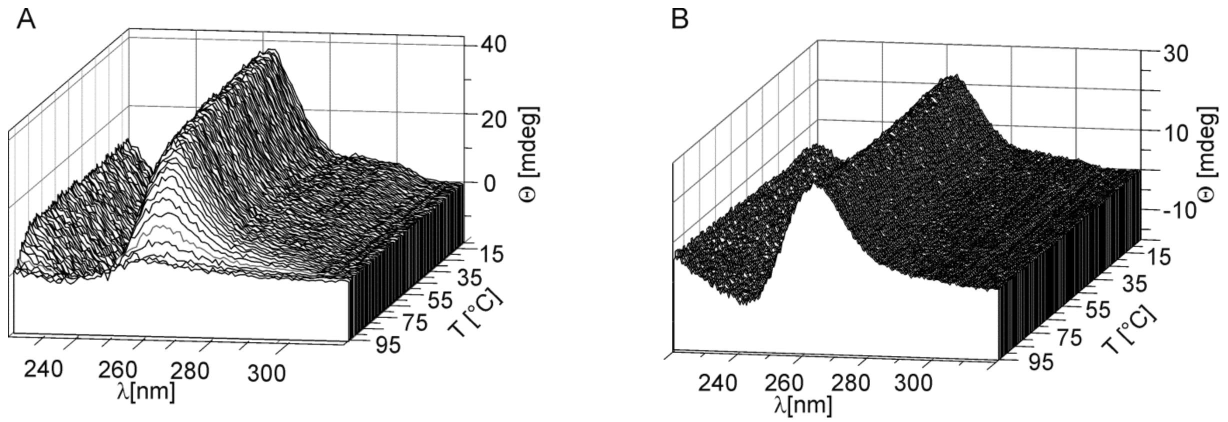

Figure S10**:** CD melting curve of d(G_4C_2) oligonucleotide in the presence of A) 100 mM $^{15}NH_4Cl$ and B) 80 mM KCl and 100 mM $^{15}NH_4Cl$. Spectra were recorded in 10 mM Li-cacodylate buffer, pH 6.0. Concentraction of oligonucleotide was 70 μM.

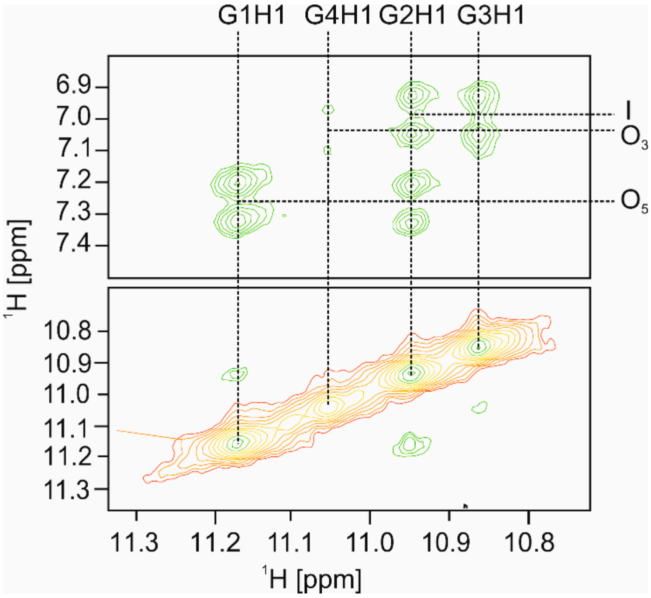

Figure S11: Imino – imino and imino - aromatic regions of ROESY spectrum (τ_m = 80 ms) of [d(G_4C_2)]$_4$. Green cross-peaks have a negative sign indicating cross-relaxation and correspond to interactions between imino protons and $^{15}NH_4^+$ ions. Vertical and horizontal dashed lines indicate resonance frequencies of imino protons and $^{15}NH_4^+$ ions at individual binding sites, respectively. Spectrum was recorded on 600 MHz NMR spectrometer, 25 °C in 90% H_2O and 10% ^2H_2O, 100 mM $^{15}NH_4Cl$, pH 6.0. Oligonucleotide concentration was 2 mM.

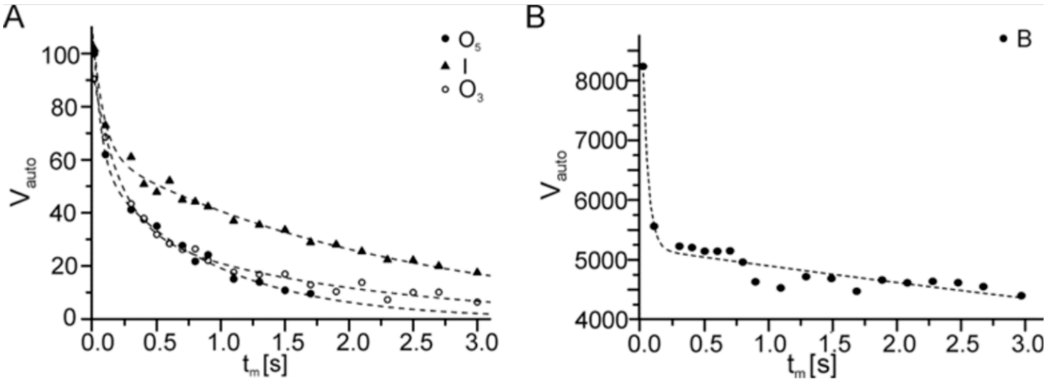

Figure S12: Relative volumes of autocorrelation peaks A) O_5 (black circles), I (black triangles) O_3 (empty circles) and B) ions in bulk solution as a function of mixing time (τ_m) at 25°C. Best fits of experimental data points to Eq. 1 are shown in black dashed curves. Parameters that best fit individual experimental data sets are listed in Table S1.

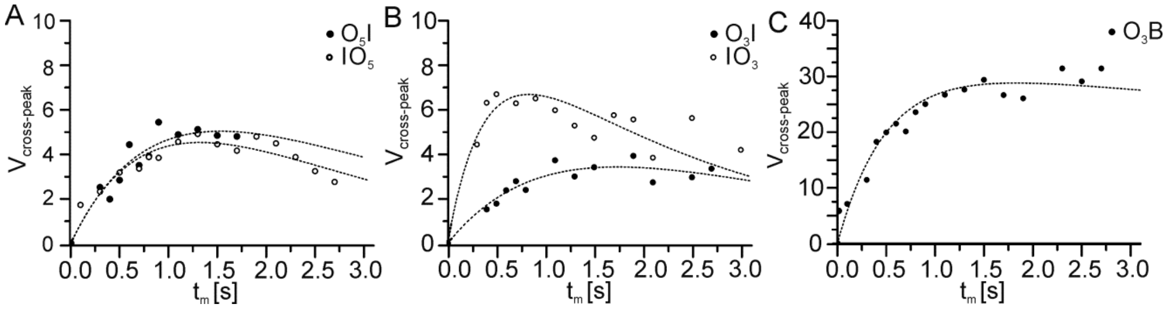

Figure S13: Relative volumes of cross- peaks A) O_5I and IO_5 shown in black and empty circles, respectively, B) O_3I and IO_3 (black and empty circles, respectively) and C) O_3B as functions of mixing time (τ_m) at 25°C. Best fits of experimental data points to Eq. 2 are shown in black dashed curves. Parameters that best fit individual experimental data sets are listed in Table S2.

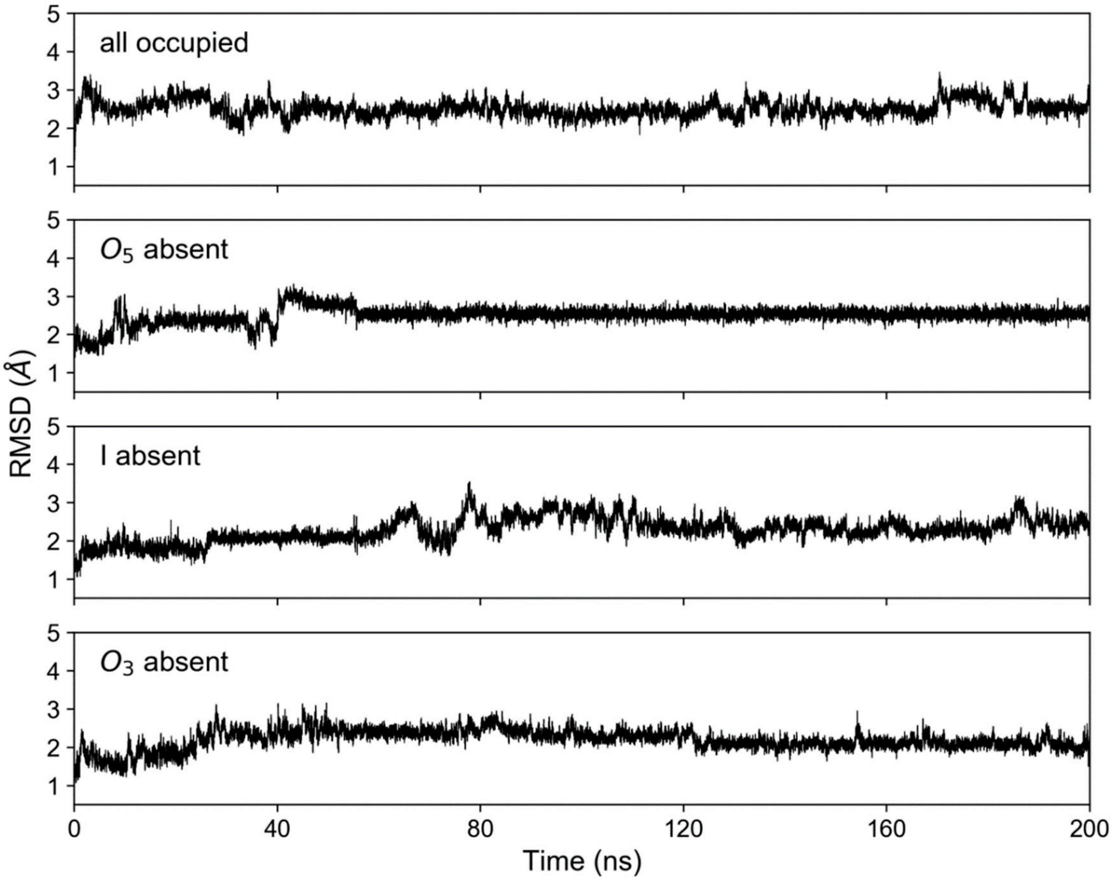

Figure S14: Backbone RMSD of the $[d(G_4C_2)]_4$ G-quadruplex in the presence of ammonium ions with respect to the starting structure from MD simulation with ammonium ions present at binding sites O_5, I and O_3, absent at binding sites O_5 I or O_3.

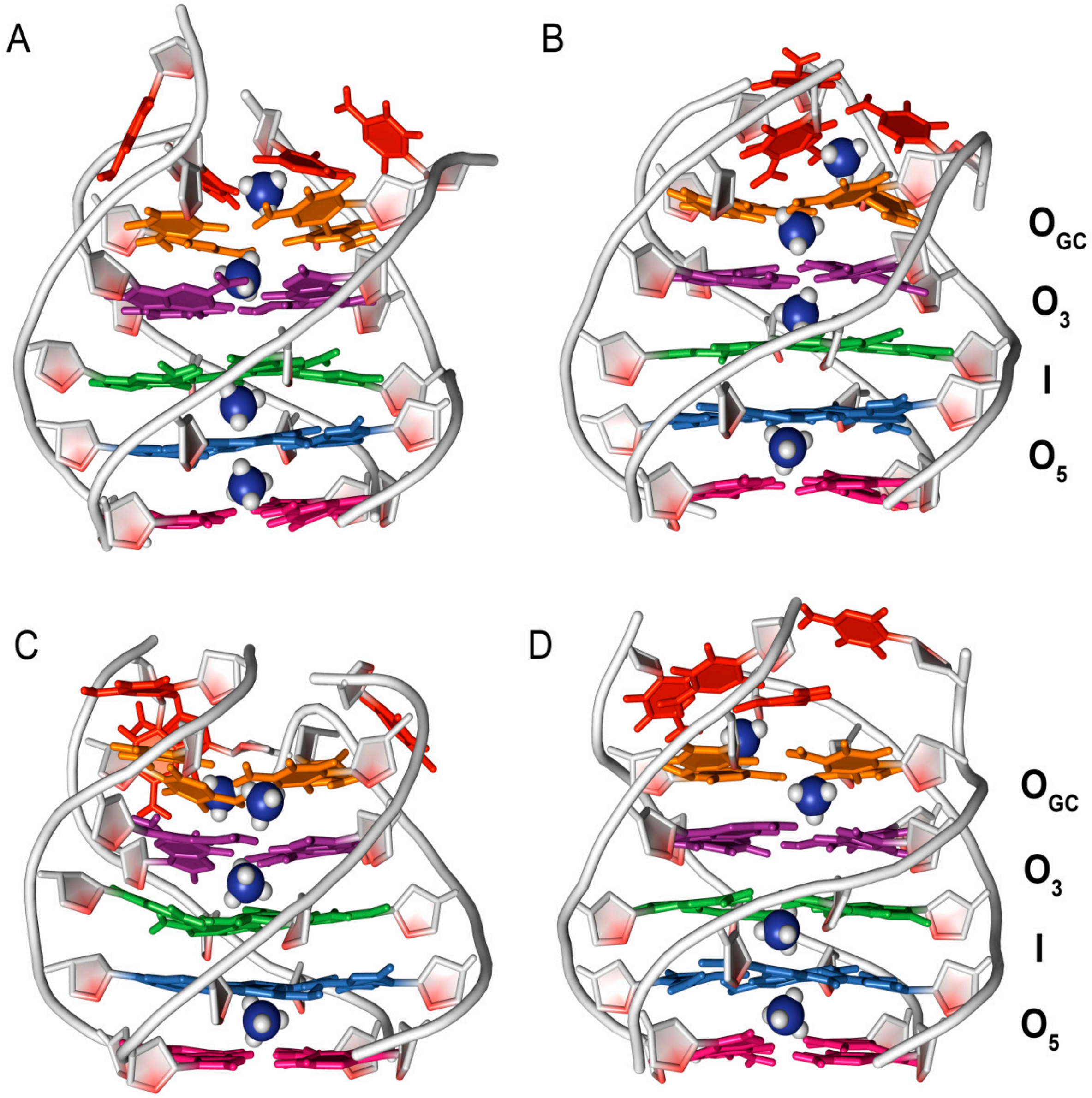

Figure S15: The structure $[d(G_4C_2)]_4$ G-quadruplex from the last frame of MD simulations with ammonium ions present in A) at binding sites O_5, I and O_3, or absent in B) at binding site O_5, C) at binding site I and D) at binding site O_3. The ammonium ion binding sites are labeled. The ammonium ions are showing in sphere model.

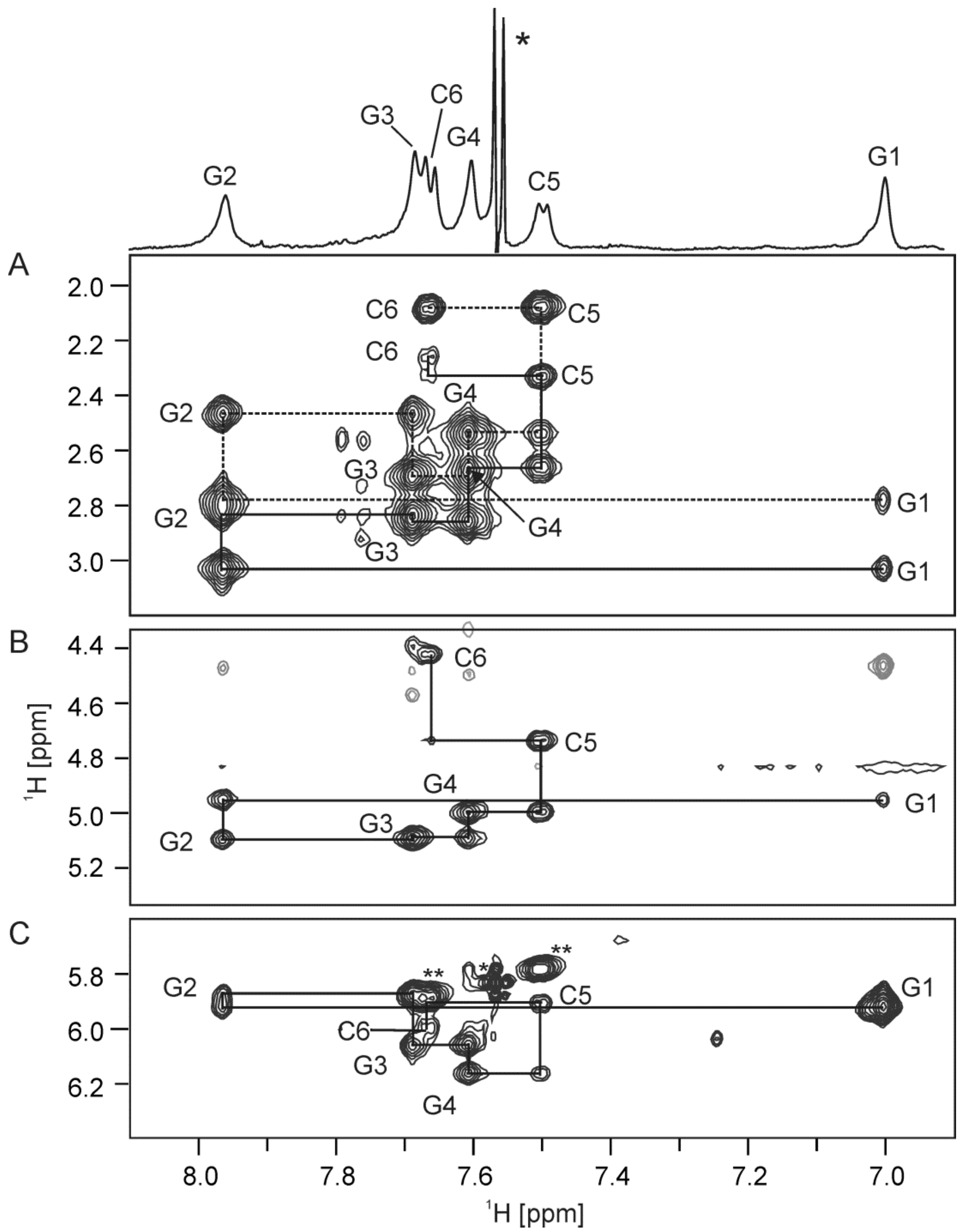

Figure S16: The aromatic H6/H8 –sugar H2'/H2'' (A), H3' (B) and H1' (C) regions of NOESY spectrum (τ_m= 150 ms) of $[d(G_4C_2)]_4$. All sequential walks except H8/H6-H2', which is shown with dashed black line, are depicted in solid black lines. Assignments are shown next to corresponding cross-peaks. Aromatic region of ^1H NMR spectrum is shown on top together with signal assignments. Signals labeled with *correspond to organic impurity while ** denotes H5-H6 cross-peaks of cytosines. Spectrum was recorded on 600 MHz NMR spectrometer, 25 °C in 90% H_2O and 10% ^2H_2O, 80 mM KCl and 100 mM $^{15}NH_4Cl$, pH 6.0. Oligonucleotide concentration was 2 mM.

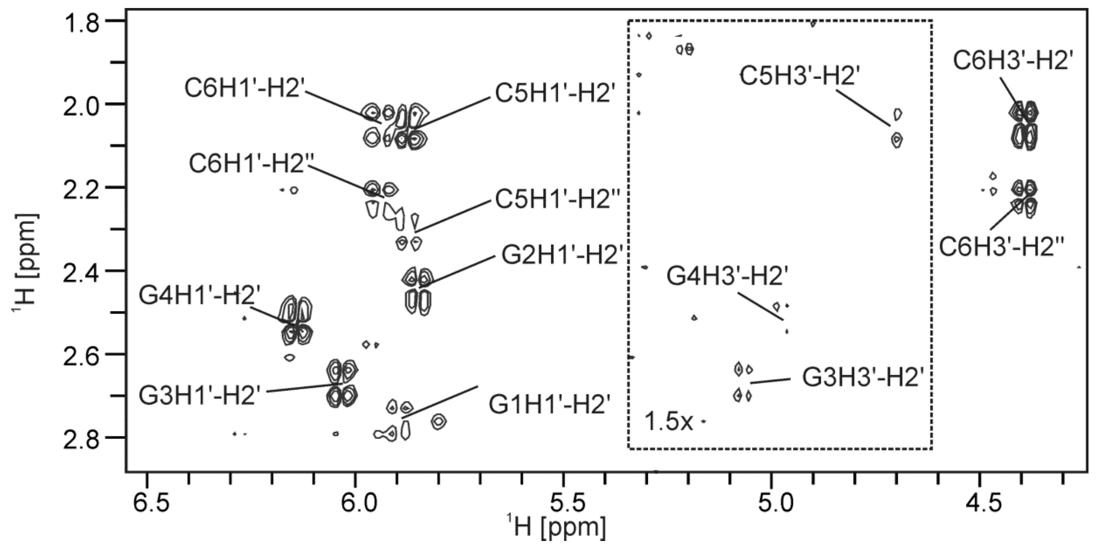

Figure S17: Sugar H1′/H3′ –sugar H2′/H2′′ region of DQF-COSY spectrum of [d(G_4C_2)]$_4$. Assignments are shown next to cross-peaks. Intensity of region indicated by black dashed square was increased by 1.5 fold in respect to the rest of region. Spectrum was recorded on 600 MHz NMR spectrometer, 25 °C in 100 % ^2H_2O, 80 mM KCl and 100 mM $^{15}NH_4Cl$, pH 6.0. Oligonucleotide concentration was 2 mM.

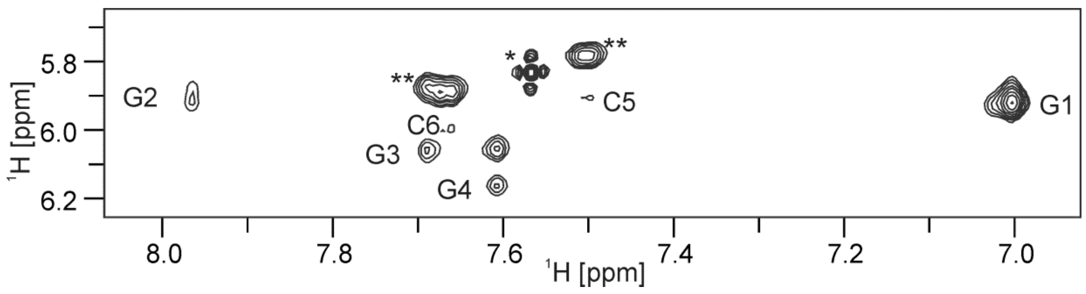

Figure S18: The aromatic H6/H8 – anomeric H1′ region of NOESY spectrum (τ_m= 80 ms) of [d(G_4C_2)]$_4$. Assignments are shown next to corresponding cross-peaks. Stronger intensity of G1H8-H1′ in comparison to cross-peaks corresponding to other residues indicates *syn* orientation around glycosidic bond for G1. Signals labeled with *correspond to organic impurity while ** denotes H5-H6 cross-peaks of cytosines. Spectrum was recorded on 600 MHz NMR spectrometer, 25 °C in 90% H_2O and 10% ^2H_2O, 80 mM KCl and 100 mM $^{15}NH_4Cl$, pH 6.0. Oligonucleotide concentration was 2 mM.

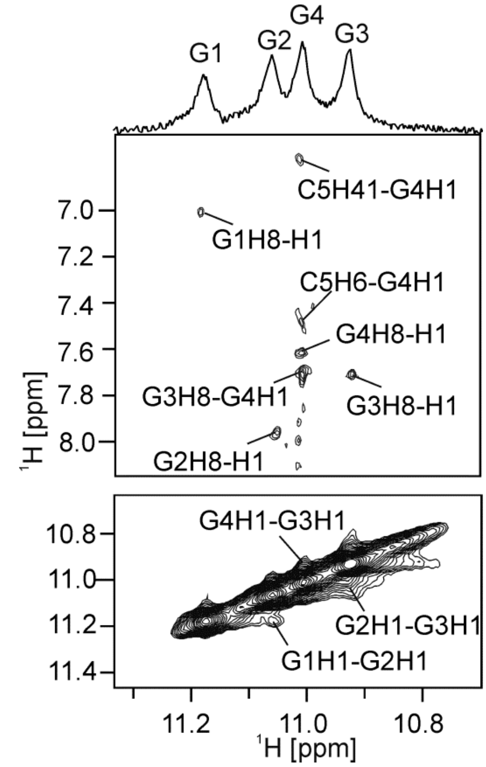

Figure S19: Imino -aromatic and imino-imino regions of NOESY spectrum (τ_m= 150 ms) of [d(G_4C_2)]$_4$. Assignments are shown next to corresponding cross-peaks. Imino region of ^{1}H spectrum is shown on top together with signal assignments. Spectrum was recorded on 600 MHz NMR spectrometer, 25 °C in 90% H_2O and 10% ^2H_2O, 80 mM KCl and 100 mM $^{15}NH_4Cl$, pH 6.0. Oligonucleotide concentration was 2 mM.

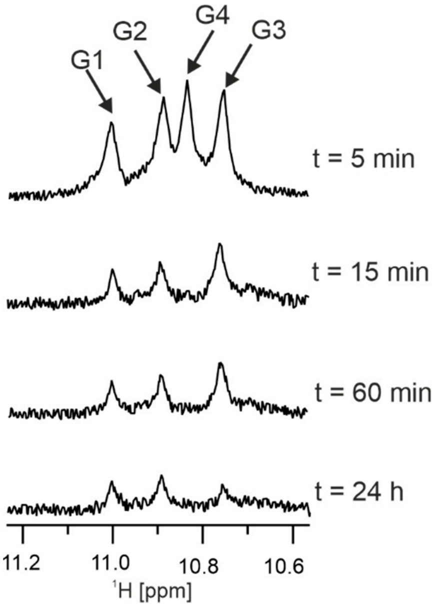

Figure S20: Imino region of ^{1}H spectra of [d(G_4C_2)]$_4$ G-quadruplex recorded at different times after dissolving into 100 % ^2H_2O. Assignments are shown next to signals. Spectra were recorded on 600 MHz NMR spectrometer, 25°C, 80 mM KCl and 100 mM $^{15}NH_4Cl$. Oligonucleotide concentration was 2 mM.

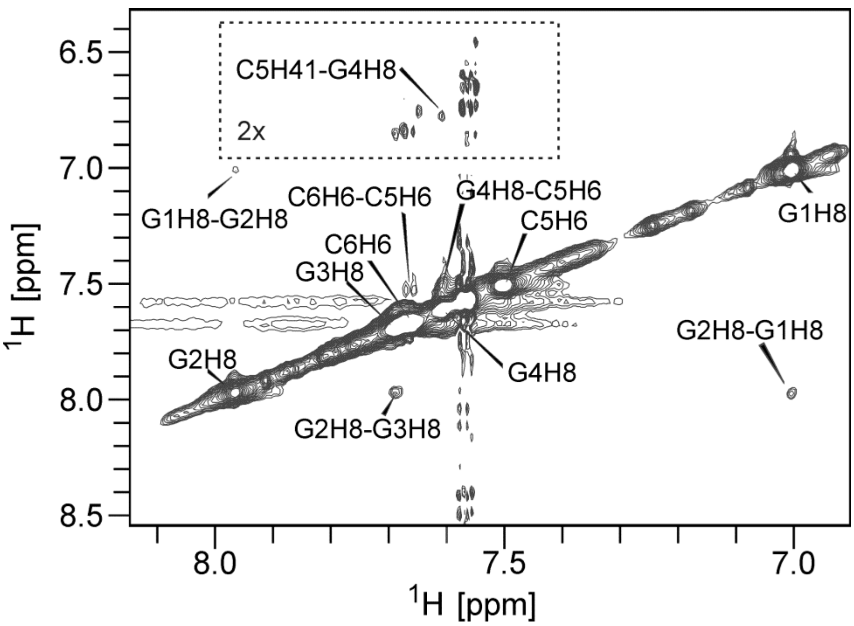

Figure S21: Aromatic region of NOESY spectrum (τ_m= 150 ms) of [d(G_4C_2)]$_4$. Assignments are shown next to corresponding cross-peaks. The aromatic protons of individual residues are noted ony with a name of the corresponding residue. Spectrum was recorded on 600 MHz NMR spectrometer, 25 °C in 90% H_2O and 10% ^2H_2O, 80 mM KCl and 100 mM $^{15}NH_4Cl$, pH 6.0. Oligonucleotide concentration was 2 mM.

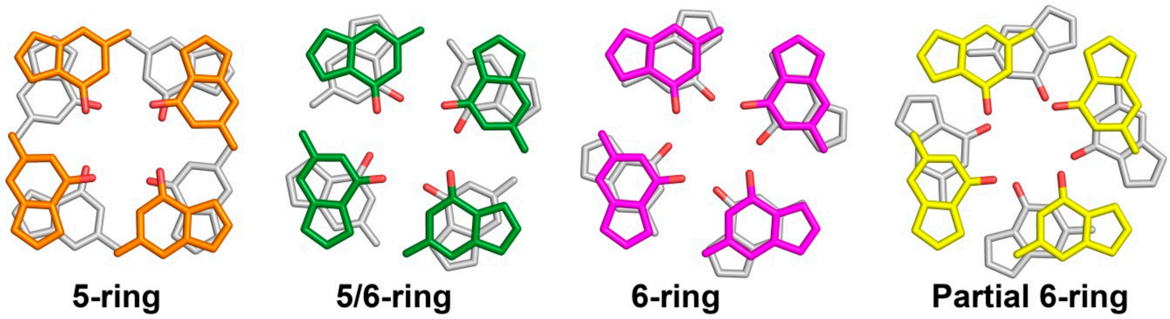

Figure S22: G-quartet base stacking modes at the interface of stacked G-quadruplexes as observed in crystallographic structures.

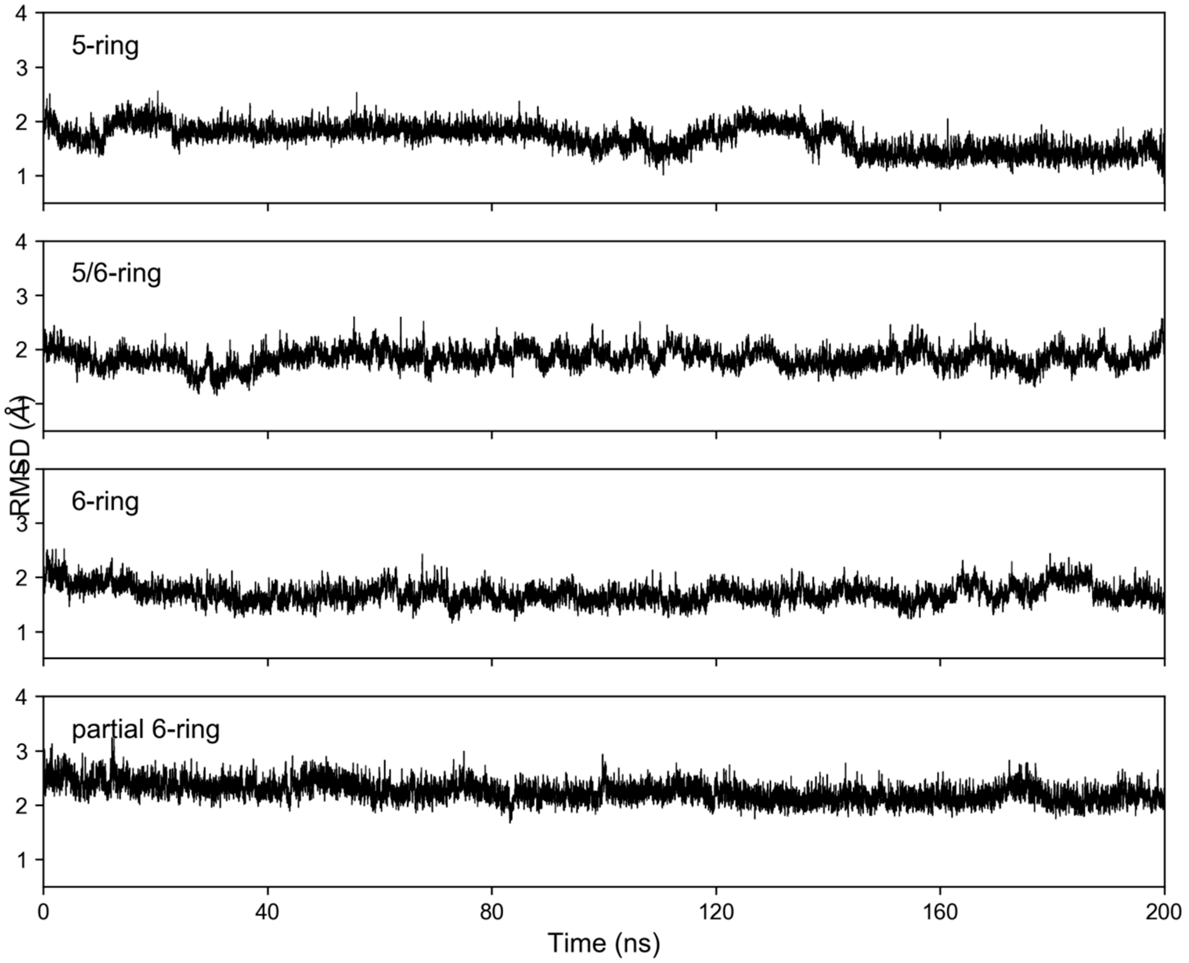

Figure S23: Backbone RMSD of the $[d(G_4C_2)]_4$ G-quadruplex dimers in K^+ ions solution with respect to the last frame of the simulation of dimer interface G-quartets initially stacking in 5-membered ring mode.

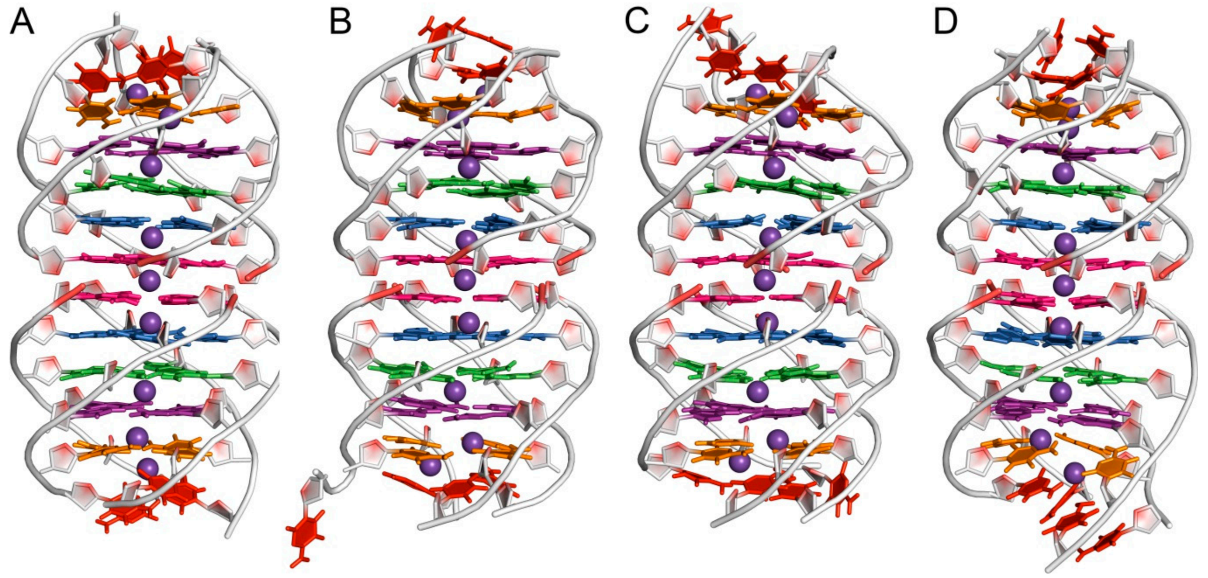

Figure S24: The structure of dimeric form of [d(G_4C_2)]$_4$ G-quadruplex from the last frame of MD simulation with dimer interface G-quartets initially stacking in A) 5-membered ring mode, B) '5/6-membered ring mode, C) 6-membered ring mode and D) partial 6-membered ring mode. The potassium ions are showing in sphere model.

SUPPLEMENTARY TABLES

Table S1: Fitting parameters for autocorrelation peaks

	O_5		I		O_3		B	
	**value**	**error**	**value**	**error**	**value**	**error**	**value**	**error**
A	58.0	2.6	62.8	1.6	57.9	3.1	4073	442
B	53.2	3.2	45.7	3.0	35.9	3.2	5317	97
T_1^{-1}	1.11	0.07	0.43	0.02	0.56	0.06	0.06	0.01
r_2	16.7	2.6	11.9	2.0	4.9	0.6	24.4	6.7
Reduced χ^2	2.59		3.96		2.68		2.55*10^10	
R^2	0.996		0.991		0.994		0.9502	

Table S2: Fitting parameters for cross peaks

	O_5I		IO_5		IO_3		O_3I		O_3B	
	**value**	**error**	**value**	**error**	**value**	**error**	**value**	**error**	**value**	**error**
A	111		109		109		94		94	
f	0.17	0.06	0.11	0.01	0.16	0.05	0.12	0.01	0.30	0.02
T_1	2.3		2.3		2.3		2.3		16.7	
k	0.5	0.2	0.8	0.2	0.3	0.1	2.1	0.4	1.6	0.3
Reduced χ^2	0.29		0.18		0.14		0.71		10.01225	
R^2	0.889		0.895		0.878		0.759		0.857	

Table S3: Structural statistics for $[d(G_4C_2)]_4$ G-quadruplexes in the presence of $^{15}NH_4Cl$ (PDB ID 8C7B) and KCl (PDB ID 8C7A).

	**$^{15}NH_4Cl$**	**KCl**
**NMR distance and torsion angle restraints**		
NOE-derived distance restraints	256	380
Intra-residue	164	236
inter-residue	92	144
Sequential	92	144
Hydrogen bond restraints	32	32
Torsion angle restraints	40	24
**Structure statistics**		
Violations		
Mean NOE restraint violation (Å)	0.1±0.1	0.1±0.1
Max. NOE restraint violation (Å)	0.184	0.184
Max. torsion angle restraint violation (°)	0.00	0.50
Pairwise heavy atom RMSD (Å)		
Overall	1.66±0.36	1.31±0.41
G1-G4	0.80±0.21	0.68±0.12
G1-C5	0.97±0.23	0.84±0.15
